# [Corrigendum] Genistein exerts growth inhibition on human osteosarcoma MG-63 cells via PPARγ pathway

**DOI:** 10.3892/ijo.2024.5635

**Published:** 2024-03-11

**Authors:** Mingzhi Song, Xiliang Tian, Ming Lu, Xianbin Zhang, Kai Ma, Zhichao Lv, Zhenxing Wang, Yang Hu, Chong Xun, Zhen Zhang, Shouyu Wang

Int J Oncol 46: 1131-1140, 2015; DOI: 10.3892/ijo.2015.2829

Subsequently to the publication of the above article, an interested reader drew to the authors' attention that, in Fig. 1D on p. 1134, the data panels showing the results for the 'Control' and '1 μmol/l GW9662' experiments (on the left hand side of the figure) were overlapping, such that these data had been derived from the same original source where they were intended to show the results from differently performed experiments. The authors were able to re-examine their original data, and realize that the data for the '1 μmol/l GW9662' panel had been selected incorrectly.

The corrected version of Fig. 1, now featuring the correct data for the '1 μmol/l GW9662' experiment in Fig. 1D, is shown on the next page, The authors confirm their error did not grossly affect either the results of the conclusions reported in the paper, and are grateful to the Editor of *International Journal of Oncology* for allowing them this opportunity to publish a Corrigendum. They also apologize to the readership for any inconvenience caused.

## Figures and Tables

**Figure 1:**
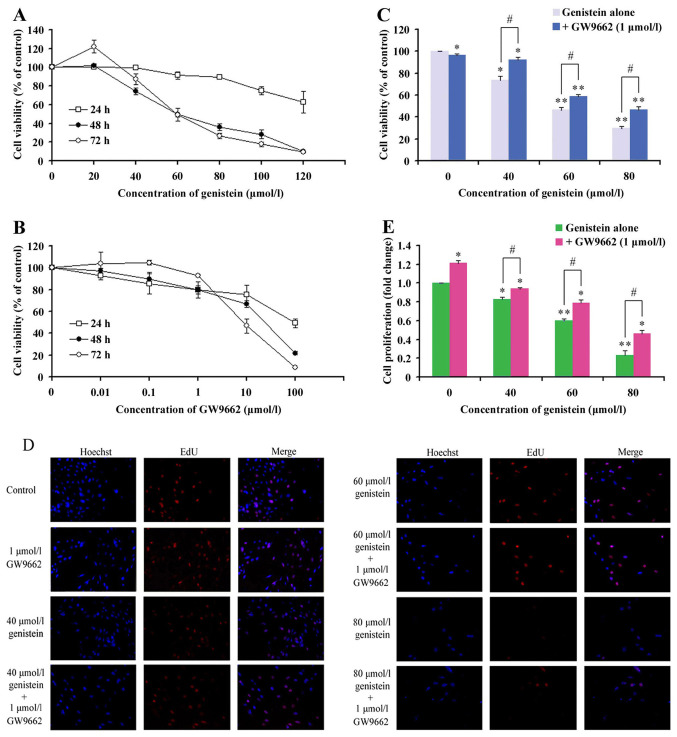
The human osteosarcoma MG-63 cell growth inhibition by genistein, GW9662 and their combination: (A) the effects of various doses of genistein on the viability of MG-63 cells after 24, 48 and 72 h; (B) the effects of various doses of GW9662 on the viability of MG-63 cells after 24, 48 and 72 h; (C) the effects of various doses of genistein with 1 μmol/l of GW9662 on the viability of MG-63 cells after 48 h. The total percentage of viable cells was measured by CCK-8 assay. Data are expressed as mean ± SD in three independent experiments. Bars marked with asterisks are significant with respect to the controls at ^*^P<0.05 and ^**^P<0.01. The number of viable cells significantly increased, compared with the relative genistein group (^#^P<0.05). EdU assay of relative Hoechst stained cells and EdU add-in cells. (D) MG-63 cells were treated with different concentrations of genistein, combination or control. Forty-eight hours after treatment, EdU (100 μmol/l) was added and the cells were cultured for 2 h. EdU and Hoechst staining were performed, as described in Materials and methods. At least 200 cells were counted per well. (E) Data are expressed as mean ± SD in the representative experiments performed in triplicate. The proliferation rate of MG-63 cells, treated with different concentrations of genistein or the combined group, significantly decreased compared with the control (^*^P<0.05). The proliferation rate of the combination group significantly increased, compared with the relative genistein group (^#^P<0.05).

